# Numerical Modelling and Analytical Comparison of Delamination during Cryogenic Drilling of CFRP

**DOI:** 10.3390/polym13223995

**Published:** 2021-11-19

**Authors:** Arunachalam S. S. Balan, Chidambaram Kannan, Kunj Jain, Sohini Chakraborty, Siddharth Joshi, Krishna Rawat, Walaa F. Alsanie, Vijay Kumar Thakur

**Affiliations:** 1 Department of Mechanical Engineering, NITK Surathkal, Mangalore 575025, India; balan@nitk.edu.in; 2 School of Mechanical Engineering, Vellore Institute of Technology, Vellore 632014, India; kunjj96@yahoo.co.in (K.J.); sohinice187@gmail.com (S.C.); siddharthjj1995@gmail.com (S.J.); drdnaa148@gmail.com (K.R.); 3 Department of Clinical Laboratories Sciences, The Faculty of Applied Medical Sciences, Taif University, P.O. Box 11099, Taif 21944, Saudi Arabia; w.alsanie@tu.edu.sa; 4 Biorefining and Advanced Materials Research Centre, SRUC, Edinburgh EH9 3JG, UK; 5 School of Engineering, University of Petroleum & Energy Studies (UPES), Dehradun 248007, India

**Keywords:** CFRP, cryogenic drilling, delamination, hole-quality, ABAQUS/CAE

## Abstract

Carbon-Fibre-Reinforced Polymers (CFRPs) have seen a steady rise in modern industrial applications due to their high strength-to-weight ratio and corrosion resistance. However, their potential is being hindered by delamination which is induced on them during machining operations. This has led to the adoption of new and innovative techniques like cryogenic-assisted machining which could potentially help reduce delamination. This study is aimed at investigating the effect of cryogenic conditions on achieving better hole quality with reduced delamination. In this paper, the numerical analysis of the drilling of CFRP composites is presented. Drilling tests were performed experimentally for validation purposes. The effects of cooling conditions and their subsequent effect on the thrust force and delamination were evaluated using ABAQUS/CAE. The numerical models and experimental results both demonstrated a significant reduction in the delamination factor in CFRP under cryogenic drilling conditions.

## 1. Introduction

Composites are having a significant influence on today’s fields of engineering and science. This is evident from their extensive applications, ranging from their use in space industries for the construction of intricate technologies such as cryogenic tanks to their use in the more conceivable fields of sports and transportation. Of all such composite materials, carbon-fibre-reinforced polymers (CFRPs) have seen a rise in popularity. This is primarily due to their high strength-to-weight ratio. The advent of CFRPs has allowed industries to use lighter and more robust materials without any loss in performance. Besides having a great strength-to-weight ratio, these materials also possess a plethora of other desirable properties such as high stiffness, low density and an excellent damping capacity. CFRPs are also immune to corrosion; however, they are prone to failure by penetration, cracking or delamination due to their highly anisotropic and inhomogeneous nature [1].

Drilling is widely used to produce holes on composite parts which are then assembled. However, due to the non-homogeneous, anisotropic and highly abradant nature of CFRPs, excessive tool wear occurs during drilling which causes fibre pullout, particle fracture and delamination [2]. Delamination is the most common form of failure in composite materials and is one of the main reasons why most of the materials (around 60%) are rejected in aircraft industries [3]. Delamination decreases the fatigue strength which, in turn, degrades the long-term performance of the composites. It also compromises the integrity of the composite layups which makes the composite unable to effectively transfer load throughout its structure. 

Extensive studies have been conducted by researchers to negate the effects of delamination. Tsao [4] conducted drilling experiments on composite materials using a step core drill. In this study, the effect of the feed and the diameter ratio on the thrust force were exhaustively investigated. A decreased diameter ratio and an increased feed were found to favour the thrust force reduction. Statistical techniques were employed to ascertain the relationship between the cutting parameters and delamination for different varieties of drills [5]. The delamination factor exhibited an increase, with a rise in both the cutting speed and feed rate.

Despite numerous models available to predict delamination, the majority of them considered the crack length alone, while a few studies conducted by Davim et al. [6] mooted the delaminated area to predict the delamination factor. A new model developed by Joshi et al. [7] that considers both the crack length and delaminated area seems to be valid for different drilling environments.

Various other ideas have been provided to minimise delamination, such as changing cutting speeds, drill bit geometries, feed rate, tool materials and tool coatings [8]. Krishnaraj et al. [9] performed high-speed drilling operations on CFRP laminates and determined the optimal machining parameters for minimal delamination and tool wear. A study on drilling bio composites revealed that a higher drill point angle induced a high thrust force [10]. Numerous techniques have been tried to reduce the damage that occurs during the drilling of polymer matrix composites. These damage reduction techniques range from using a backup plate at the drill exit site to the adoption of variable feed rates in which the feed rate was substantially reduced as the drill approached the hole exit [11]. The drilling performed on graphite/bismaleimide composite laminates with high-speed steel, carbide and diamond tools disclosed the supremacy of diamond tools in achieving the least surface damage and delamination [12].

Numerous literatures are available on the machining of CFRPs concerning tool wear, the effect of drilling parameters and the variability of the machining conditions. Bhattacharyya et al. [13] conducted cryogenic machining studies on Kevlar composites. The development of high thrust forces and low tool wear were observed when machining was done in the presence of liquid nitrogen. However, a limited amount of research is available on cryogenic drilling. Cryogenic drilling experiments conducted on Kevlar laminates demonstrated the occurrence of a high thrust force with a reduced temperature [14]. Drilling under cryogenic conditions also exhibited a great improvement in the overall quality of the hole [15]. 

Simulation software such as Abaqus possesses high computing ability and is quite easy to perform. It also allows for the numerical validation of various proposed loading models [16]. Phapale et al. [17] conducted drilling simulations using FEM analysis software to study the high-speed drilling mechanism of CFRPs, and an acceptable deviation of 5% between experimental and simulation results was reported. Shan et al. [18] performed three-dimensional numerical simulations on 2.5D carbon/carbon (C/C) composites and further proposed a damage initiation model based on Hashin’s failure criterion and the Shokrieh–Lessard model. About six failure modes were considered in the model to predict the material damage and failure for different feeds. Wang et al. [19] performed 3D finite element modelling of the CFRP drilling using Abaqus/CAE, while Kendrew et al. [20] developed an accurate model for CFRP ripping to aid in the design and fine-tuning of the assembly before prototyping. The main objectives of the present work are formulated as (i) an experimental assessment of the delamination factor and thrust force under dry and cryogenic drilling on CFRPs, (ii) numerical and analytical modelling of CFRP drilling under the identical operating conditions using Abaqus/CAE and (iii) a comparative evaluation of the results obtained through experiments and numerical simulation.

## 2. Numerical Modelling 

### 2.1. Drill Geometry

A 3D CAD model of the tungsten carbide drill with a diameter of 8 mm was designed in SolidWorks with the specifications provided by the tool manufacturer. The drill had a helix angle of 30°. The tool geometry had to be very precise to reduce any errors and to obtain accurate simulation results. To reduce the computational time, the drill bit was cut by 40 mm from the lower tip. The 3D model was then imported in Abaqus and was later assembled with the workpiece model for the simulation.

**Figure 1:**
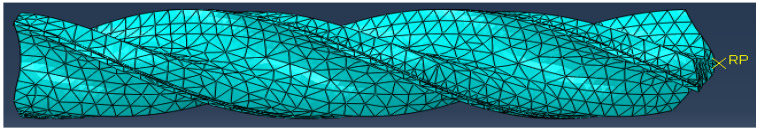
A three-dimensional model of the meshed drill bit.

For this simulation, the tool was assumed to be a rigid and nondeformable body, while the workpiece was modelled as a 3D deformable and rigid body. This assumption is justified as the yield stress of tungsten carbide is considerably higher than that of the CFRP. No material property was assigned to the drill bit as it was treated as a rigid body for the simulation. This is shown in Figure 1.

### 2.2. Modelling Aspects

The three major aspects of modelling the simulation were the boundary conditions, the interaction properties and the meshing. The boundary conditions were given to simulate the experimental procedure with as much accuracy as possible. The drill was assembled in the system in such a way that the tip of the drill was 2 mm above the workpiece centre. The drill was made to move in the –Z direction as per the setup. The drill movement was restricted in the X and Y directions, and it was given translational velocity as well as rotational velocity in the –Z direction. All the boundary conditions for the drill were assigned using the velocity boundary conditions on a singular reference point on the drill. The total period of the entire simulation was given as 2.5 s to reduce the computational load. Long step times generally lead to excessively long calculations and at the same time provide erroneous outcomes and convergence issues.

Two major interactions occurred in the model: viz. 1. the interaction between the composite workpiece layers and 2. the interaction between workpiece layers and the drill. The cohesive elements of the composite layers of the workpiece are the main ones to show delamination. The CSDMG parameter is used in the field output variables to see where the nodal damage represents the scalar stiffness degradation for cohesive surfaces. A wedge with a free structure arrangement was used for the meshing of the drill because of its delicate geometry. The element size was given as 1 mm to have a fine mesh that does not cause high distortions.

## 3. Material Modelling

### 3.1. Mechanical Properties

The mechanical properties of the CFRP were implemented in the simulation using the property module in Abaqus. Table 1 provides the mechanical properties of the CFRP which were used for the dry drilling simulation, while the properties used for the simulation of the cryogenic-assisted CFRP drilling were obtained from the literature [21,22].

**Figure 2:**
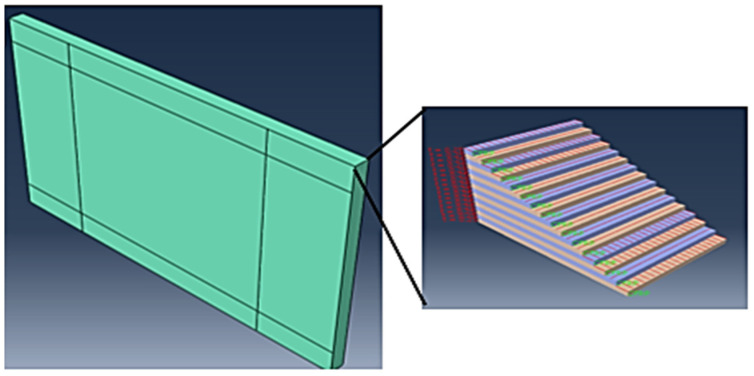
Representation of the ply plot.

The 30 mm × 50 mm workpiece was modelled in Abaqus/CAE. The CFRP workpiece was modelled with two fibre orientations (0° and 90°) in a 21-layer setup by using the composite layup option in Abaqus. The properties of the 3D deformable solid in the extrusion mode were applied to the workpiece. To simulate the composite characteristics, cohesive elements were added to the model with the cohesive surface parameters. The CFRP ply stack plot is shown in Figure 2.

### 3.2. Modelling Aspects of CFRP

**Figure 3:**
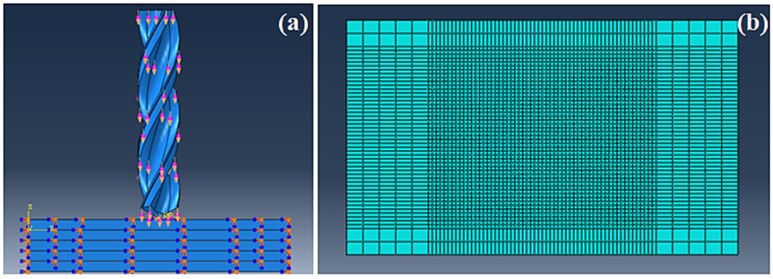
(**a**) Boundary conditions (**b**) Meshed model.

The boundary condition for the CFRP was set with the purpose of clamping the workpiece from all directions. All movements were restricted for the workpiece using the encastre boundary conditions on the side faces of the CFRP layers. Different interactions were given for cryogenic and dry drilling. The cryogenic model takes the heat transfer interaction between the drill and the workpiece into consideration as the liquid nitrogen was sprayed on the tip of the drill and the uppermost layer of the CFRP for the entire duration of drilling. In the simulation, this boundary condition was given by giving the conductivity values of the CFRP in material properties. Further, the CFRP layers were given a predefined temperature of −196 °C to simulate the cooling due to liquid nitrogen. The boundary conditions and the meshed model are portrayed in Figure 3.

Reduced-integrated 8-node shell elements were used for the meshing of the CFRP plate. Cohesive behaviour was given between the plies for the simulation. The mesh density around the drilled hole was considered up to the element size of 0.05 mm to reduce the computational cost and to optimise the results for a clearer picture of the delaminated area. The element deletion criterion was activated to delete any excessively damaged elements. This is an important step as undeleted elements cause computational errors and problems during visualization. A finer mesh was given to the area which was facing damage, while the ends of the workpiece were given a coarser mesh size. The mesh was a hex element shaped to mesh with a swept structure, which allowed the mesh to be even on both the front and backside of the workpiece. An advanced front meshing algorithm was used as per the default setting in the software. An SC8R mesh was used during the thermal analysis as the use of a thermal shell created problems in the convergence of the simulation. The detailed specifications of both the workpiece and tool meshing are listed in Table 2.

### 3.3. Failure Criteria 

Hashin’s failure criteria were employed as the primary failure criteria as they assume four failure modes for composite materials; viz. 1. fibre tension, 2. fibre compression, 3. matrix tension and 4. matrix compression.

Fibre tension *σ*_11_ ≥ 0

(1) $${\left(\frac{\sigma_{11}}{X_{T}}\right)}^{2}+\frac{\sigma_{12}{}^{2}+\sigma_{13}{}^{2}}{S_{12}^{2}}=\geq 1\;failure<1\;nofailure$$

Fibre compression *σ*_11_ < 0

(2) $$\;\;{\left(\frac{\sigma_{11}}{X_{c}}\right)}^{2}=\geq 1\;failure<1\;nofailure$$

Matrix tension *σ*_22_ + *σ*_33_ > 0

(3) $$\frac{{\left(\sigma_{22}+\sigma_{33}\right)}^{2}}{Y_{T}^{2}}+\frac{\sigma_{23}{}^{2}+\sigma_{22}\sigma_{33}}{S_{23}^{2}}+\frac{\sigma_{12}{}^{2}+\sigma_{13}{}^{2}}{S_{12}^{2}}=\geq 1\;failure<1\;nofailure$$

Matrix compression *σ*_22_ + *σ*_33_ > 0

(4) $$\left[{\left(\frac{Y_{c}}{2S_{23}}\right)}^{2}-1\right]\left(\frac{\sigma_{22}+\sigma_{33}}{Y_{C}}\right)+\frac{{\left(\sigma_{22}+\sigma_{33}\right)}^{2}}{4S_{23}^{2}}+\frac{\sigma_{23}{}^{2}+\sigma_{22}\sigma_{33}}{S_{23}^{2}}+\frac{\sigma_{12}{}^{2}+\sigma_{13}{}^{2}}{S_{12}^{2}}=\geq 1\;failure<1\;nofailure$$

The damage evolution of the CFRP was based on the energy dissipation during the process and is linear according to the Abaqus built-in Hashin model. The various modes and their corresponding failure criteria for the fibre and matrix are presented by Equations (1)–(4). In Abaqus/CAE, the element is deleted when the damage variables reach the value of 1 or greater under the failure modes presented in the above equations. The value of the variables viz. DAMAGEFT (fibre tensile damage variable), DAMAGEFC (fibre compressive damage variable), DAMAGEMT (matrix tensile damage variable) and DAMAGEMC (matrix compressive damage variable) is checked, and if the value is greater than the threshold, the element will be deleted according to the Hashin model subroutine incorporated in Abaqus/Standard CAE. Hashin’s parameters are well established and widely adopted in the industries [23].

#### Damage Initiation Criteria

Before machining or initiating any damage, the material property is assumed to be linear elastic for CFRP composite laminates. Thus, the orthotropic elastic material is modelled with the stress–strain relationship.

(5) $$\Gamma =1/\left(v_{12}v_{21}-v_{23}v_{32}-v_{13}v_{31}-2v_{21}v_{32}v_{13}\right)$$

$C_{11}^{0}=E_{1}\left(1-v_{23}v_{32}\right)\Gamma$

$C_{22}^{0}=E_{2}\left(1-v_{13}v_{31}\right)\Gamma$

$C_{33}^{0}=E_{3}\left(1-v_{12}v_{21}\right)\Gamma$

$C_{12}^{0}=E_{1}\left(v_{21}+v_{31}v_{23}\right)\Gamma$

$C_{23}^{0}=E_{2}\left(v_{32}+v_{12}v_{31}\right)\Gamma$

$C_{13}^{0}=E_{1}\left(v_{31}+v_{21}v_{32}\right)\Gamma$

$C_{44}^{0}=G_{12}$

$C_{55}^{0}=G_{23}$

$C_{66}^{0}=G_{13}$

Once the damage initiation criterion is satisfied, the material degradation will start taking place. The two coefficients of the damage will come into the equation (df and dm). The coefficients will change accordingly.


(6) $$d_{f}=-1-\left(1-d_{ft}\right)\left(1-d_{fc}\right)$$

(7) $$\;d_{m}=-1-\left(1-d_{mt}\right)\left(1-d_{mc}\right)$$

$C_{11}^{0}=\left(1-d_{f}\right)E_{1}\left(1-v_{23}v_{32}\right)\Gamma$

$C_{22}^{0}=\left(1-d_{f}\right)\left(1-d_{m}\right)E_{2}\left(1-v_{13}v_{31}\right)\Gamma$

$C_{33}^{0}=\left(1-d_{f}\right)\left(1-d_{m}\right)E_{3}\left(1-v_{12}v_{21}\right)\Gamma$

$C_{12}^{0}=\left(1-d_{f}\right)\left(1-d_{m}\right)E_{1}\left(v_{21}+v_{31}v_{23}\right)\Gamma$

$C_{23}^{0}=\left(1-d_{f}\right)\left(1-d_{m}\right)E_{2}\left(v_{32}+v_{12}v_{31}\right)\Gamma$

$C_{13}^{0}=\left(1-d_{f}\right)\left(1-d_{m}\right)E_{1}\left(v_{31}+v_{21}v_{32}\right)\Gamma$

$C_{44}^{0}=\left(1-d_{f}\right)\left(1-s_{mt}d_{mt}\right)E_{1}\left(1-s_{mc}d_{mc}\right)G_{12}$

$C_{55}^{0}=\left(1-d_{f}\right)\left(1-s_{mt}d_{mt}\right)E_{1}\left(1-s_{mc}d_{mc}\right)G_{23}$

$C_{66}^{0}=\left(1-d_{f}\right)\left(1-s_{mt}d_{mt}\right)E_{1}\left(1-s_{mc}d_{mc}\right)G_{13}$

## 4. Methodology

**Figure 4:**
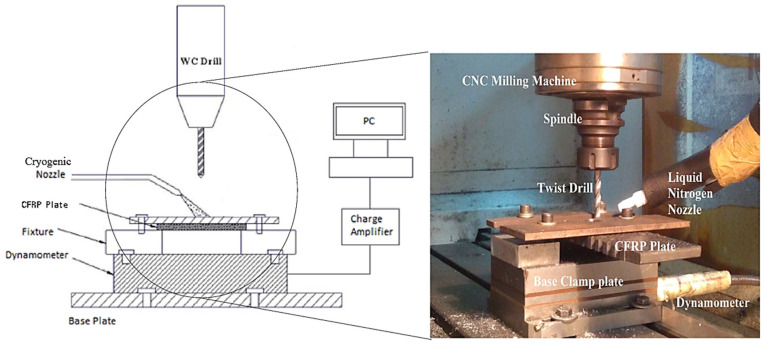
Experimental setup.

A computer numerical controlled (CNC) vertical drilling machine (Model: SURYA VF30, BFW—India, Bangalore, India) was used for conducting the experiments. The drill used was an 8 mm solid carbide drill (uncoated) from Kennametal with a point angle of 130°. Solid carbide drills have excellent thermal stability, which was of prime importance while drilling under cryogenic conditions. After each experiment, a new drill bit was used to minimise errors due to tool wear. The experimental setup adopted in this research is presented in Figure 4.

The drilling procedure was performed conventionally, while the liquid nitrogen was sprayed at the tool–workpiece interaction. This was done to maximise the utilisation of the cryogenic fluid. The workpiece was clamped between custom-made fixtures, which had sufficient room for the cryogenic fluid to affect a substantial area. After the workpiece was firmly secured, holes were drilled through its entire depth. The dynamometer (Kistler 9257B) was fixed on the mild steel base and was insulated using foam to protect it from subzero temperatures. A nozzle was used to spray the liquid nitrogen on the workpiece at 1 bar pressure and −196 °C. Drilling was done both in a cryogenic and dry environment.

## 5. Results and Discussion

### 5.1. Analysis of Delamination

**Figure 5:**
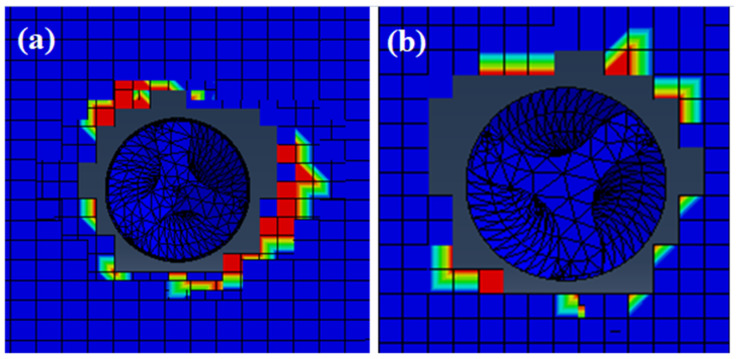
(**a**) Entry side hole delamination (**b**) Exit side hole delamination.

Delamination is the most common form of damage in CFRPs. A lot of research has been done to quantify the damage caused by delamination. Generally, delamination is expressed by a parameter known as the delamination factor (D_f_). The delamination at the exit and entry sides of the drilled hole is shown in Figure 5a and Figure 5b, respectively.

Traditionally, it is defined as the ratio of the largest delamination diameter to the drilled hole diameter.

(8) $$\;\;D_{f}=\frac{D}{d}$$

The ratio of the delaminated circle area to the drilled hole area is also sometimes referred to as the delamination factor.

(9) $$D_{f}=\frac{A}{a}$$

An analytical model for assessing the delamination factor during the cryogenic drilling of the CFRP has been developed by the authors and elaborately detailed in previously published work [7]. This model introduces a factor that takes into account the thrust force. It is a function of the maximum thrust force to the critical thrust force.

(10) $$D_{f}=w_{1}\frac{D_{max}}{D_{0}}+w_{2}\frac{A_{max}}{A_{0}}+w_{3}\frac{F_{max}}{F_{crit}}$$

In the present work, the delamination around the holes is studied using experimental, numerical and analytical methods. An ultrasonic C-scanner is used for assessing the delamination under the experimental approach, while the delamination assessment under numerical modelling is carried out by Abaqus/CAE. Later, the analytical approach makes use of Equation (10) to assess the delamination. The delamination at the tool exit area is primarily considered as it faces the most damage. The delamination as determined by the experimental, analytical and numerical approaches under a dry and cryogenic environment is presented in Table 3.

It is inferred from the experiments that the cutting speed and feed rate have a considerable influence on the delamination factor. The delamination factor was found to be least at 125 m/min and 0.03 mm/rev under dry drilling conditions. A visual representation of the delaminated holes in both dry and cryogenic conditions is presented in Table 4. It also shows the experimental images which were obtained from the ultrasonic C-scanner.

**Figure 6:**
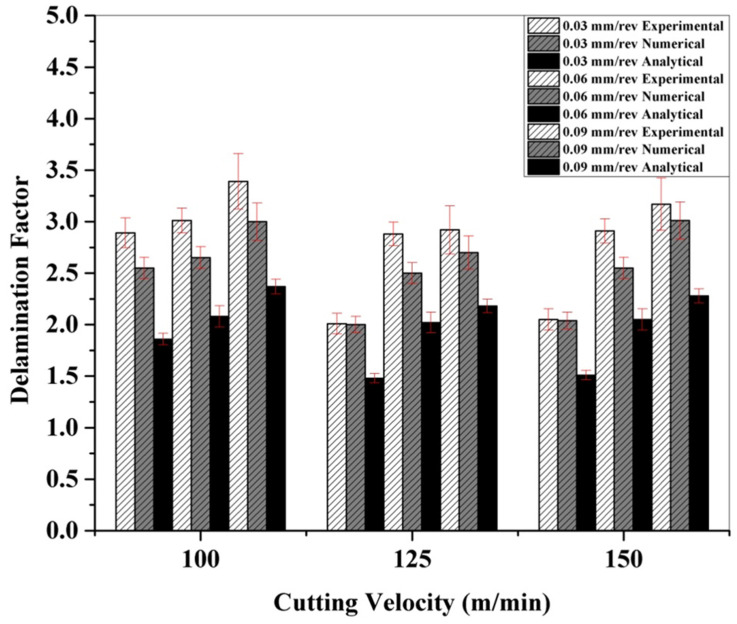
Experimental and numerical delamination factors under dry drilling of CFRP.

The influence of the feed rate and cutting velocity on the delamination factors under the dry drilling of CFRP is presented in Figure 6. The results of the other two approaches, namely, analytical and simulation are also portrayed for comparison and validation purposes. When the cutting velocity is maintained constant, the delamination factor was found to worsen with increased feed rates. The same trend was observed under all the different approaches investigated in the present research work. High magnitude thrust forces developed under high feed rates cause considerable damage to the CFRP matrix. This, in turn, increases the delamination factor [7]. With increasing cutting speed, the delamination factor was found to decrease and beyond certain velocities, it was found to increase again. Higher cutting speeds result in high temperatures which soften the CFRP matrix and thus reduce delamination. However, the resistance to delamination was decreased beyond a certain threshold temperature, which might be associated with an increased delamination factor at higher cutting speeds (>125 m/min) [24].

The delamination factor values as predicted by the numerical model were found to be closer to the experimental values (within 15%). However, the values obtained using the analytical model showed greater deviation from the experimental values as compared to the numerical data. This is because $\frac{F_{max}}{F_{crit}}$ has less impact on the overall delamination factor than that of $\frac{D_{max}}{D_{0}}$ [7].

**Figure 7:**
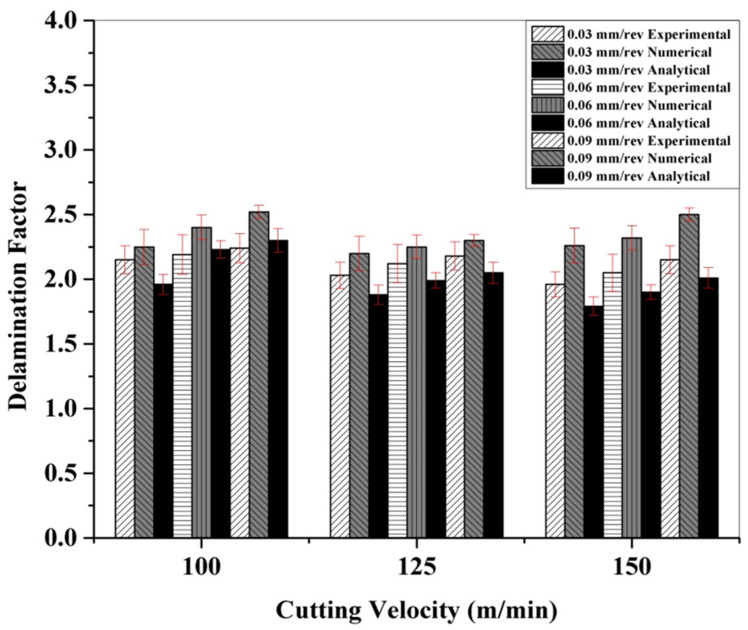
Experimental and numerical delamination factors under cryogenic drilling of CFRP.

The influence of the feed rate and cutting velocity on the delamination factors under cryogenic-assisted CFRP drilling is presented in Figure 7. As observed in dry drilling, in cryogenic drilling, the delamination factor was also found to increase with increased feed rates at a constant cutting speed. But their values were considerably lower than that of dry drilling. The numerical and analytical results followed the same trend of the experimental outcomes. This can be inferred from Figure 7. A reduced delamination factor at increasing cutting speeds and fixed feed rates is attributed to the reduction in the thrust force, which leads to less damage induced on the CFRP [7].

The values of the delamination factor predicted by the numerical model were very close to the experimental values (within 15%). The values obtained using the analytical model were relatively close to the experimental values. Under a cryogenic environment, a higher impact is established by $\frac{F_{max}}{F_{crit}}$ on the delamination factor than $\frac{D_{max}}{D_{0}}$ on account of a higher thrust force and a lower probability of crack formation in the cryogenic environment [7].

### 5.2. Analysis of Force

From Table 5, we can infer that the magnitude of the thrust forces under cryogenic conditions is considerably higher. This is attributed to the induced compressive stress due to the difference in the thermal expansion coefficients of resin and fibre under cryogenic conditions [15]. Thus, the elastic modulus and the tensile strength of the CFRP are further augmented.

**Figure 8:**
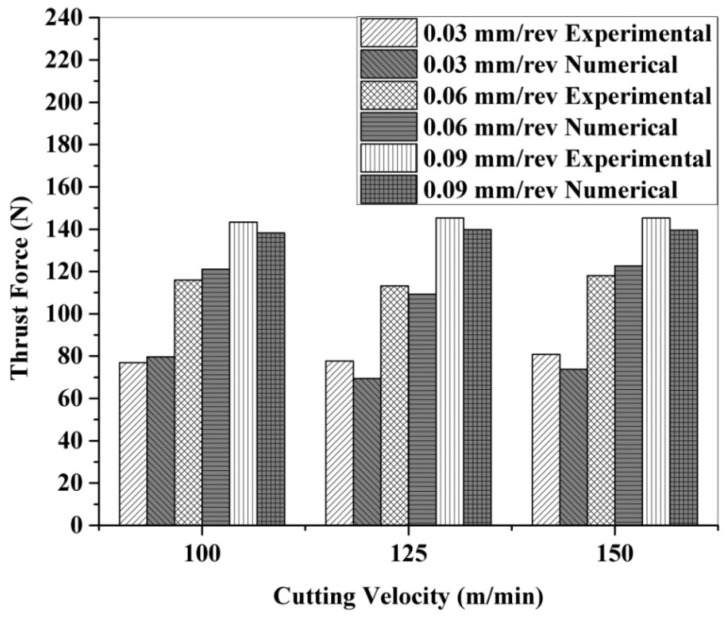
Experimental and numerical thrust force under dry drilling of CFRP.

The experimental and numerical predicted thrust force for CFRP drilling in a dry environment under different operating parameters is shown in Figure 8. The trend of an increased thrust force with increasing feed rates at a constant cutting speed might be associated with the reduction in the clearance angle [25]. The cutting speed has very little effect on the thrust forces because the mechanism of cutting in composites is based on bending fracture. Due to work hardening, high magnitude thrust forces are developed with increasing cutting speeds in metals. However, this phenomenon is not observed in composite laminates [26,27,28]. The experimental value is found to be in the range of 77–146 N for dry drilling. In addition, the numerically predicted results are found to be in the acceptable range (within 10%) of experimental observations.

**Figure 9:**
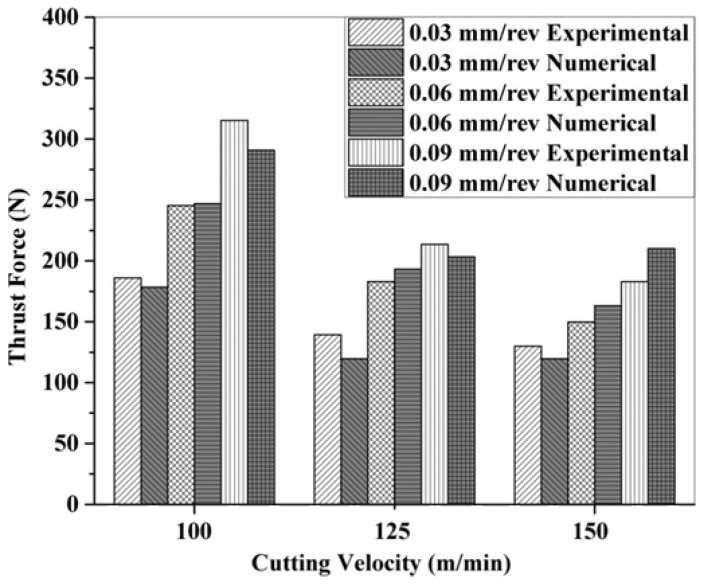
Experimental and numerical thrust force under cryogenic drilling of CFRP.

The experimental and numerical results of the thrust force for CFRP drilling under a cryogenic environment with varying feed rates and cutting speeds are shown in Figure 9. The same increasing trend is observed under experimental and numerical predictions as in the case of dry drilling. However, the observed thrust forces are higher than dry drilling. Yet, in contrast, a decreasing trend is observed with increasing cutting speeds. This is associated with the migration of the failure mode from bending to shear fracture due to the reduced fibre deflection in cryogenic drilling [7]. The experimental value is found to be in the range of 139–183 N for cryogenic conditions. The values of the thrust force predicted by the numerical model were very close to the experimental values (within 14%).

### 5.3. Surface Morphology Studies

**Figure 10:**
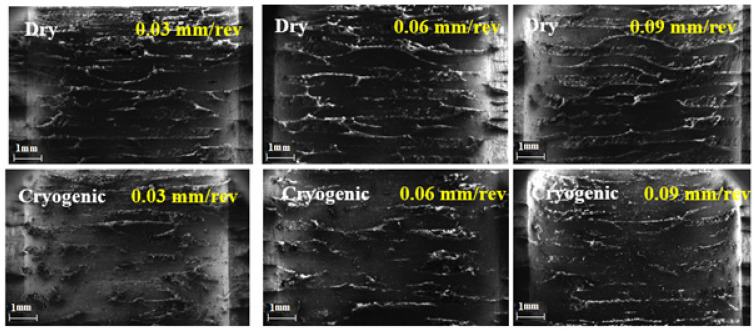
Scanning electron microscopic images of drilled holes with different feed rates under dry and cryogenic conditions.

The surface morphology of the drilled holes at a higher cutting velocity (150 m/min) under dry and cryogenic conditions is shown in Figure 10.

In dry drilling, the matrix gets decomposed and softened due to higher cutting temperatures, resulting in poor surface quality. Under cryogenic conditions, better surface quality with fewer fibre pullouts is seen. Even though the thrust force is higher under a cryogenic environment, the surface quality deterioration is significantly less when compared with dry drilling. This is mainly due to the increase in fibre brittleness caused by liquid nitrogen. Thus, the failure mode of the fibre under cryogenic drilling changes from rupture to shear fracture. As the feed rate increases, the surface quality deteriorates. With increasing feed, the uncut chip thickness increases, resulting in higher order cutting forces. A high magnitude cutting force increases the angle between the fibre orientation and cutting direction, thus increasing the fibre pullout on the drilled surface.

## 6. Conclusions

An exhaustive investigation was carried out to study the effects of the cryogenic environment on the thrust force and delamination factor during CFRP drilling. The present study that combines the experimental, numerical and analytical modelling approaches stands as evidence for the success of cryogenic conditions in mitigating the drilling-induced delamination of CFRP. This study closely predicts the values of the delamination factor as well as its variation with the changes in operating parameters. The major outcomes of the present study are summarised and presented below:

- The thrust force obtained in cryogenic drilling is found to be much larger than that in dry drilling. An increase in the feed rate leads to increased thrust forces; the same trend is observed under the simulation and the experimentation as well.
- The delamination factor in cryogenic drilling is found to be much lower than that in dry drilling. An increase in the feed rate results in an increase in the delamination factor. This is ensured by both the experimentation and simulation results.
- The delamination factor obtained via the numerical model is in good agreement with both the experimental and force-adjusted delamination factors for cryogenic drilling.
- Drilling in cryogenic conditions gives better results than dry drilling that is evident from the experimental and simulation results.

The experimental, numerical and analytical modelling exhibited the supremacy of cryogenic over the dry condition for CFRP drilling. The reduced delamination factor will reduce the rejection rates in aerospace and other industries, which utilise CFRP for manufacturing a wide variety of components. The present research work can be extended by the numerical and analytical modelling of CFRP drilling under sustainable environments such as chilled air and minimum quantity lubrication (MQL).

## Figures and Tables

**Table 1:** List of factors and levels considered for the regression analysis.

Property	Value
Elastic modulus, E_11_ (GPa)	127
Elastic modulus, E_22_ = E_33_ (GPa)	9.1
Poisson’s ratio, ν_12_ = ν_13_	0.31
Poisson’s ratio, ν_23_	0.45
Shear modulus, G_12_ = G_13_ (GPa)	5.6
Shear modulus, G_23_ (GPa)	4
Density, ρ (kg/m^3^)	1600

**Table 2:** Mesh parameters.

Mesh Parameters	Workpiece	Drill
No. of Elements	3264	10,545
Mesh Size	0.5	1
Element Code	SC8R	CD10M
Element Type	Continuum Shell	3D Stress
Element Shape	Hex	Tet
Technique	Sweep	Free
Element Deletion	Yes	Default
Second Order Accuracy	No	No
Max Degradation	0.01	Default
Element Library	Standard	Explicit
Geometric Order	Linear	Quadratic

**Table 3:** Delamination determination by different approaches.

Speed (m/min)	Feed (mm/rev)	Dry Environment			Cryogenic Environment		
		Exp.	Numerical	Analytical	Exp.	Numerical	Analytical
100	0.03	2.89	2.55	1.88	2.15	2.25	2.0
100	0.06	3.01	2.65	2.11	2.19	2.4	2.28
100	0.09	3.39	3	2.4	2.24	2.52	2.35
125	0.03	2.05	2.05	1.5	2.03	2.20	1.92
125	0.06	2.88	2.25	2.04	2.12	2.25	2.03
125	0.09	2.92	2.30	2.21	2.18	2.3	2.09
150	0.03	2.05	2	1.53	1.96	2.26	1.75
150	0.06	2.91	2.55	2.08	2.05	2.32	1.94
150	0.09	3.17	3.01	2.32	2.15	2.50	2.05

**Table 4:** Image of drilled holes (Experimental, FEM, Optical).

Speed (m/min)	Feed (mm/rev)	Dry Environment			Cryogenic Environment		
		Exp.	FEM	Optical	Exp.	FEM	Optical
100	0.09	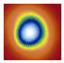	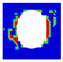	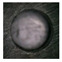	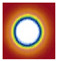	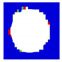	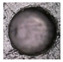
0125	0.09	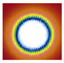	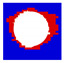	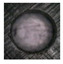	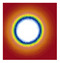	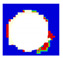	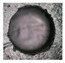
150	0.09	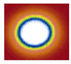	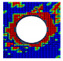	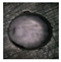	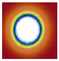	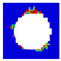	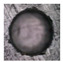

**Table 5:** Experimental and numerical results of the thrust force.

Iteration	Speed (m/min)	Feed (mm/rev)	Cryogenic Environment		Dry Environment	
			Exp.	Numerical	Exp.	Numerical
1	100	0.03	186	178.4	76.9	79.64
2	100	0.06	245.4	246.9	115.9	121.05
3	100	0.09	315.4	290.9	143.4	138.2
4	125	0.03	139.4	119.7	77.7	69.38
5	125	0.06	183	193.3	113.2	109.24
6	125	0.09	213.6	203.4	145.3	139.81
7	150	0.03	129.9	119.7	80.9	73.81
8	150	0.06	149.7	163.2	118.1	122.6
9	150	0.09	183.1	210.1	145.3	139.6

## Data Availability

Data can be made available upon request.

## Nomenclature

*σ*_ij_	stress in i-j plane
*ε*_ij_	strain in i-j plane
Co	Coefficients
*E*_i_	Young’s modulus in i direction
*G*_ij_	Shear modulus in i-j plane
*ν*_ij_	Poisson’s ratio (for transverse strain in the j direction when stress is in the i direction)
*d_f_*	Damage coefficient for fibre
*d_m_*	Damage coefficient of the matrix
*D_f_*	Delamination factor
S_ij_	Allowable shear strengths (i = 1, 2 or 3)
X_t_ and Y_t_	Allowable tensile strengths
X_c_ and Y_c_	Allowable compressive strengths
X_1t_	Tensile strength in the longitudinal direction
X_2t_	Tensile strength in the transverse direction
X_1c_	Compressive strength in the longitudinal direction
X_2c_	Compressive strength in the longitudinal direction
12S	Shear strength in the longitudinal direction
